# Aqua­(benzamidato-κ*N*)bis­[3,5-difluoro-2-(pyridin-2-yl)phenyl-κ*C* ^1^]iridium(III) methanol monosolvate

**DOI:** 10.1107/S1600536812005922

**Published:** 2012-02-17

**Authors:** Songlin Zhang, Feng Wu, Yuqiang Ding

**Affiliations:** a School of Chemical and Material Engineering, Jiangnan University, Wuxi 214122, Jiangsu Province, People’s Republic of China

## Abstract

In the title compound, [Ir(C_11_H_6_F_2_N)_2_(C_7_H_6_NO)(H_2_O)]·CH_3_OH, the Ir^III^ ion adopts an octa­hedral geometry, and is coordinated by two 3,5-difluoro-2-(pyridin-2-yl)phenyl ligands, one mol­ecule of water and one benzamidate anion. The two 2-(4,6-difluoro­phen­yl)pyridyl ligands are arranged in a *cis*-*C*,*C′* and *trans*-*N*,*N′* fashion. Additionally, there is a bystanding methanol mol­ecule outside the coordination sphere of the Ir^III^ ion. In the crystal, mol­ecules of the title compound are linked by O—H⋯O and O—H⋯N hydrogen bonds. One F atom of each ligand is equally disordered over two sites. The C atom of the solvent molecule is likewise disordered over two sites in a 0.589 (11):0.411 (11) ratio.

## Related literature

For related cyclo­metallated Ir^III^ complexes containing a *κ* ^2^-bound benzaminate anion, see: Yang *et al.* (2011 ▶); Wang *et al.* (2008 ▶); Zhang *et al.* (2011 ▶). For the coordination geometry of some homoleptic meridional and heteroleptic iridium(III) complexes, see: Tamayo *et al.* (2003 ▶); Yang *et al.* (2007 ▶); You & Park (2005 ▶); Zhang *et al.* (2011 ▶). For the general procedure of preparing a chloride-bridged Ir^III^ dimer, see: Nonoyama (1974 ▶).
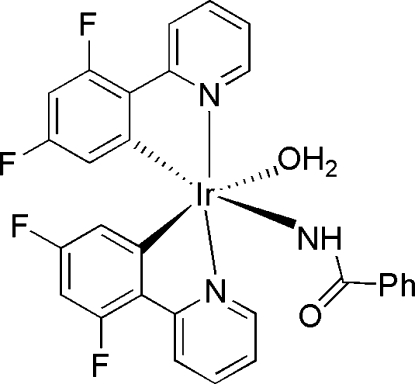


## Experimental

### 

#### Crystal data

[Ir(C_11_H_6_F_2_N)_2_(C_7_H_6_NO)(H_2_O)]·CH_4_O
*M* *_r_* = 742.74
Monoclinic, 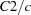

*a* = 29.544 (4) Å
*b* = 11.6258 (12) Å
*c* = 20.247 (3) Å
β = 129.391 (2)°
*V* = 5374.5 (12) Å^3^
*Z* = 8
Mo *K*α radiation
μ = 5.04 mm^−1^
*T* = 223 K
0.34 × 0.25 × 0.24 mm

#### Data collection

Rigaku Saturn diffractometer
Absorption correction: multi-scan (*REQAB*; Jacobson, 1998 ▶) *T*_min_ = 0.279, *T*_max_ = 0.378
14977 measured reflections
6122 independent reflections
5277 reflections with *I* > 2σ(*I*)
*R*_int_ = 0.031

#### Refinement

*R*[*F* ^2^ > 2σ(*F* ^2^)] = 0.045
*wR*(*F* ^2^) = 0.101
*S* = 1.09
6122 reflections
392 parameters
4 restraints
H atoms treated by a mixture of independent and constrained refinement
Δρ_max_ = 1.20 e Å^−3^
Δρ_min_ = −1.18 e Å^−3^

### 

Data collection: *CrystalClear* (Rigaku, 2007 ▶); cell refinement: *CrystalClear*; data reduction: *CrystalClear*; program(s) used to solve structure: *SHELXS97* (Sheldrick, 2008 ▶); program(s) used to refine structure: *SHELXL97* (Sheldrick, 2008 ▶); molecular graphics: *SHELXTL* (Sheldrick, 2008 ▶); software used to prepare material for publication: *SHELXTL*.

## Supplementary Material

Crystal structure: contains datablock(s) I, global. DOI: 10.1107/S1600536812005922/br2185sup1.cif

Structure factors: contains datablock(s) I. DOI: 10.1107/S1600536812005922/br2185Isup2.hkl

Additional supplementary materials:  crystallographic information; 3D view; checkCIF report

## Figures and Tables

**Table 1:** Hydrogen-bond geometry (Å, °)

*D*—H⋯*A*	*D*—H	H⋯*A*	*D*⋯*A*	*D*—H⋯*A*
O2—H2*B*⋯N3	0.85 (1)	2.54 (6)	3.029 (7)	117 (6)
O2—H2*B*⋯O1	0.85 (1)	1.74 (3)	2.560 (6)	161 (7)
O2—H2*A*⋯N1	0.85 (1)	2.45 (6)	2.960 (6)	119 (6)
O2—H2*A*⋯O1^i^	0.85 (1)	1.97 (4)	2.700 (7)	142 (6)

Symmetry code: (i) 
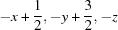
.

**Fig. 1.**
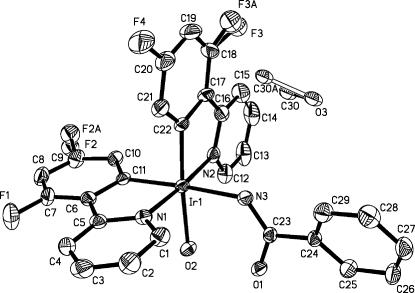
Molecular drawing of the title compound at 30% probability level. Hydrogen atoms are omitted for clarity. There are some disorder for the two fluorine atoms bound to C9 and C18 of phenyl rings.

**Fig. 2.**
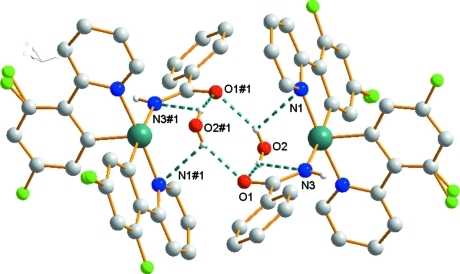
Hydrogen bonding.

## supplementary crystallographic information

### Comment

The title compound was obtained unexpectedly during our study on the preparation of bis(2-(4,6-difluorophenyl)pyridyl)iridium(III) *κ*^2^-benzamidate complex. Ancillary *κ*^2^-amidate ligands have been shown to have a great influence on the photophysical and electrochemical properties of cyclometalated Ir(III) complexes (Yang *et al.*, 2011; Wang *et al.*, 2008). We have synthesized several such bis(2-(4,6-difluorophenyl)pyridyl)iridium(III) *κ*^2^-amidate complexes, in all of which, there is a substitutent on the amide nitrogen atom (Zhang *et al.*, 2011). When benzamide lacking a substituent on the amide nitrogen atom was subjected to the reaction condition for the preparation of Ir(III) *κ*^2^-amidate complexes, an unexpected Ir(III) complex with a *N*-bound benzamidate ligand and an *O*-bound water ligand, *i.e.*, the title compound, was obtained. As shown in Fig. 1, the iridium center adopts an octahedral geometry. The two 2-(4,6-difluorophenyl)pyridyl ligands are arranged in a *cis*-*C*, *C'* and *trans*-*N*, *N'* fashion, which have already been observed in some homoleptic meridional iridium(III) complexes (Tamayo *et al.*, 2003) and also some heteroleptic iridium(III) complexes (Yang *et al.*, 2007; You & Park, 2005; Zhang *et al.*, 2011). The remaining two coordination sites were occupied by benzamidate nitrogen atom and water oxygen atom, respectively. The methanol molecule outside the coordination sphere of the Ir center should result from the deprotonation of the benzamide ligand by the intermediate Ir-OMe complex, which is generated by reaction of chloro-bridged Ir(III) dimer with sodium methoxide. However, there is still some uncertainties about the nature of the bystanding solvent molecule or how it is arranged. In the crystal, there should be intermolecular hydrogen bonds, such as O-H-O and N-H-O, as shown by Fig. 2.

### Experimental

Into a Schlenk tube containing chloro-bridged bis(2-(4,6-difluorophenyl)pyridyl) Ir(III) dimer (1 eq.), benzamide (2.5 eq.) and sodium methoxide (10 eq.) was added dichloromethane solvent under dinitrogen. The chloro-bridged Ir(III) dimer was obtained by reaction of IrCl_3_^.^3H_2_O with 2-(4,6-difluorophenyl)pyridine ligand in ethoxyethanol solvent under dinitrogen atmosphere according to the general procedure reported by Nonoyama (1974). The mixture was stirred for 48 h at room temperature, resulting in the formation of an orange solution. The CH_2_Cl_2_ solvent in the crude product mixture was then evaporated and the residue was then washed by dried ether. The crystal of the title compound was obtained by recrystallization of the solid in CH_2_Cl_2_/cyclohexane mixed solvent.

### Refinement

Hydrogen atoms bound to carbon atoms were positioned geometrically with C—H = 0.93 Å or 0.96 Å and refined using a riding model, with *U*iso(H) = 1.2*U*eq(C) or 1.5*U*eq(C). Hydrogen atoms bound to nitrogen and oxygen atoms were located in difference maps and refined subject to a restraint of N—H = 0.87 (2) Å and O—H = 0.85 (2) Å respectively. The methanol molecule in the crystal should come from the deprotonation of benzamide ligand by intermediate Ir(III)-OMe complex, which is supposed to be generated by transmetalation of chloro-bridged Ir(III) dimer with added sodium methoxide. Due to the disorder of the methanol molecule, the positions of hydrogen atoms on methanol are difficult to determine. Furthermore, this would lead to some disorder in the positions of fluorine atoms on the phenyl ring because there should be some interaction between the methanol hydrogen atoms with the fluorine atoms. This is believed to be the most probable structure of the title compound.

### Crystal data

**Table:**

[Ir(C_11_H_6_F_2_N)_2_(C_7_H_6_NO)(H_2_O)]·CH_4_O	*F*(000) = 2960
*M**_r_* = 742.74	*D*_x_ = 1.846 Mg m^−^^3^
Monoclinic, *C*2/*c*	Mo *K*α radiation, λ = 0.71075 Å
Hall symbol: -C 2yc	Cell parameters from 11541 reflections
*a* = 29.544 (4) Å	θ = 3.1–27.5°
*b* = 11.6258 (12) Å	µ = 5.04 mm^−^^1^
*c* = 20.247 (3) Å	*T* = 223 K
β = 129.391 (2)°	Block, yellow
*V* = 5374.5 (12) Å^3^	0.34 × 0.25 × 0.24 mm
*Z* = 8	

### Data collection

**Table:**

Rigaku Saturn diffractometer	6122 independent reflections
Radiation source: fine-focus sealed tube	5277 reflections with *I* > 2σ(*I*)
Graphite monochromator	*R*_int_ = 0.031
Detector resolution: 14.63 pixels mm^-1^	θ_max_ = 27.5°, θ_min_ = 3.2°
ω scans	*h* = −38→30
Absorption correction: multi-scan (REQAB; Jacobson, 1998)	*k* = −15→12
*T*_min_ = 0.279, *T*_max_ = 0.378	*l* = −21→26
14977 measured reflections	

### Refinement

**Table:**

Refinement on *F*^2^	Primary atom site location: structure-invariant direct methods
Least-squares matrix: full	Secondary atom site location: difference Fourier map
*R*[*F*^2^ > 2σ(*F*^2^)] = 0.045	Hydrogen site location: inferred from neighbouring sites
*wR*(*F*^2^) = 0.101	H atoms treated by a mixture of independent and constrained refinement
*S* = 1.09	*w* = 1/[σ^2^(*F*_o_^2^) + (0.0407*P*)^2^ + 26.4741*P*] where *P* = (*F*_o_^2^ + 2*F*_c_^2^)/3
6122 reflections	(Δ/σ)_max_ = 0.002
392 parameters	Δρ_max_ = 1.20 e Å^−^^3^
4 restraints	Δρ_min_ = −1.18 e Å^−^^3^

### Special details

**Table:**

Geometry. All e.s.d.'s (except the e.s.d. in the dihedral angle between two l.s. planes) are estimated using the full covariance matrix. The cell e.s.d.'s are taken into account individually in the estimation of e.s.d.'s in distances, angles and torsion angles; correlations between e.s.d.'s in cell parameters are only used when they are defined by crystal symmetry. An approximate (isotropic) treatment of cell e.s.d.'s is used for estimating e.s.d.'s involving l.s. planes.
Refinement. Refinement of *F*^2^ against ALL reflections. The weighted *R*-factor *wR* and goodness of fit *S* are based on *F*^2^, conventional *R*-factors *R* are based on *F*, with *F* set to zero for negative *F*^2^. The threshold expression of *F*^2^ > σ(*F*^2^) is used only for calculating *R*-factors(gt) *etc*. and is not relevant to the choice of reflections for refinement. *R*-factors based on *F*^2^ are statistically about twice as large as those based on *F*, and *R*- factors based on ALL data will be even larger.

### Fractional atomic coordinates and isotropic or equivalent isotropic displacement parameters (Å^2^)

**Table:**

	*x*	*y*	*z*	*U*_iso_*/*U*_eq_	Occ. (<1)
Ir1	0.347048 (9)	0.650175 (18)	−0.014534 (14)	0.03168 (9)	
F1	0.4503 (2)	1.0543 (4)	0.0772 (3)	0.0836 (15)	
F2	0.3963 (8)	0.9191 (12)	−0.1797 (9)	0.054 (4)	0.50
F2A	0.4011 (16)	0.913 (3)	−0.175 (2)	0.170 (12)	0.50
F3	0.438 (3)	0.306 (5)	−0.067 (4)	0.073 (8)	0.50
F3A	0.451 (3)	0.326 (5)	−0.049 (4)	0.089 (12)	0.50
F4	0.5660 (2)	0.4827 (5)	0.2118 (4)	0.0969 (18)	
O1	0.22923 (18)	0.5920 (4)	−0.0254 (3)	0.0452 (11)	
O2	0.26439 (19)	0.7445 (4)	−0.0750 (3)	0.0416 (10)	
H2A	0.279 (3)	0.774 (6)	−0.027 (2)	0.050*	
H2B	0.249 (3)	0.688 (4)	−0.070 (5)	0.050*	
N1	0.3828 (2)	0.7418 (4)	0.0941 (3)	0.0371 (11)	
N2	0.3159 (2)	0.5635 (4)	−0.1235 (3)	0.0385 (11)	
N3	0.3141 (2)	0.5160 (4)	0.0160 (3)	0.0368 (11)	
H3	0.3352	0.4537	0.0359	0.044*	
C1	0.3830 (3)	0.7075 (6)	0.1581 (4)	0.0487 (16)	
H1	0.3678	0.6348	0.1550	0.058*	
C2	0.4049 (3)	0.7770 (8)	0.2280 (5)	0.063 (2)	
H2	0.4051	0.7507	0.2720	0.076*	
C3	0.4262 (3)	0.8823 (8)	0.2333 (5)	0.063 (2)	
H3A	0.4402	0.9305	0.2801	0.075*	
C4	0.4271 (3)	0.9189 (6)	0.1684 (4)	0.0505 (17)	
H4	0.4421	0.9918	0.1713	0.061*	
C5	0.4058 (3)	0.8471 (5)	0.1000 (4)	0.0402 (14)	
C6	0.4042 (3)	0.8707 (5)	0.0273 (4)	0.0382 (13)	
C7	0.4257 (3)	0.9693 (6)	0.0165 (4)	0.0493 (16)	
C8	0.4244 (3)	0.9879 (6)	−0.0520 (5)	0.0551 (18)	
H8	0.4395	1.0551	−0.0575	0.066*	
C9	0.3994 (3)	0.9010 (7)	−0.1115 (5)	0.0535 (18)	
C10	0.3775 (3)	0.8013 (6)	−0.1057 (4)	0.0411 (14)	
H10	0.3616	0.7445	−0.1482	0.049*	
C11	0.3791 (2)	0.7851 (5)	−0.0359 (4)	0.0331 (12)	
C12	0.2627 (3)	0.5836 (6)	−0.1998 (4)	0.0524 (17)	
H12	0.2385	0.6403	−0.2034	0.063*	
C13	0.2430 (4)	0.5236 (7)	−0.2718 (5)	0.070 (2)	
H13	0.2058	0.5387	−0.3242	0.084*	
C14	0.2788 (5)	0.4403 (8)	−0.2660 (6)	0.078 (3)	
H14	0.2661	0.3972	−0.3143	0.093*	
C15	0.3329 (4)	0.4215 (7)	−0.1894 (6)	0.069 (2)	
H15	0.3578	0.3667	−0.1856	0.083*	
C16	0.3516 (3)	0.4824 (6)	−0.1166 (5)	0.0498 (17)	
C17	0.4074 (3)	0.4732 (6)	−0.0308 (5)	0.0468 (16)	
C18	0.4528 (4)	0.3966 (7)	−0.0035 (6)	0.063 (2)	
C19	0.5056 (4)	0.3973 (7)	0.0764 (7)	0.072 (3)	
H19	0.5354	0.3450	0.0929	0.086*	
C20	0.5129 (3)	0.4784 (7)	0.1314 (6)	0.065 (2)	
C21	0.4706 (3)	0.5553 (6)	0.1116 (5)	0.0520 (17)	
H21	0.4780	0.6080	0.1527	0.062*	
C22	0.4169 (3)	0.5537 (5)	0.0303 (4)	0.0408 (14)	
C23	0.2672 (2)	0.5124 (5)	0.0096 (4)	0.0353 (13)	
C24	0.2570 (3)	0.4127 (5)	0.0461 (4)	0.0359 (13)	
C25	0.2003 (3)	0.3869 (6)	0.0129 (5)	0.0505 (16)	
H25	0.1690	0.4317	−0.0319	0.061*	
C26	0.1894 (4)	0.2950 (7)	0.0454 (6)	0.065 (2)	
H26	0.1507	0.2774	0.0221	0.078*	
C27	0.2346 (4)	0.2312 (7)	0.1105 (6)	0.063 (2)	
H27	0.2271	0.1691	0.1321	0.076*	
C28	0.2916 (4)	0.2567 (7)	0.1455 (5)	0.062 (2)	
H28	0.3227	0.2124	0.1908	0.075*	
C29	0.3025 (3)	0.3481 (6)	0.1133 (5)	0.0506 (17)	
H29	0.3413	0.3661	0.1376	0.061*	
O3	0.44588 (19)	0.2099 (4)	0.2396 (3)	0.0543 (12)	
C30	0.4370 (5)	0.2603 (9)	0.1702 (7)	0.0543 (12)	0.589 (11)
C30A	0.4817 (6)	0.2785 (10)	0.2269 (12)	0.0543 (12)	0.411 (11)

### Atomic displacement parameters (Å^2^)

**Table:**

	*U*^11^	*U*^22^	*U*^33^	*U*^12^	*U*^13^	*U*^23^
Ir1	0.03266 (13)	0.03359 (13)	0.03432 (13)	0.00037 (10)	0.02386 (11)	0.00088 (10)
F1	0.097 (4)	0.068 (3)	0.087 (3)	−0.037 (3)	0.059 (3)	−0.021 (3)
F2	0.087 (10)	0.054 (6)	0.046 (6)	−0.033 (7)	0.054 (7)	−0.011 (5)
F2A	0.18 (3)	0.22 (3)	0.18 (2)	−0.02 (2)	0.15 (2)	0.03 (2)
F3	0.10 (2)	0.048 (10)	0.11 (2)	−0.003 (11)	0.09 (2)	−0.026 (12)
F3A	0.10 (2)	0.07 (2)	0.11 (2)	0.010 (16)	0.07 (2)	−0.021 (15)
F4	0.047 (3)	0.089 (4)	0.114 (4)	0.020 (3)	0.032 (3)	0.024 (3)
O1	0.038 (2)	0.042 (2)	0.063 (3)	0.004 (2)	0.035 (2)	0.006 (2)
O2	0.037 (2)	0.046 (3)	0.047 (2)	0.004 (2)	0.029 (2)	0.009 (2)
N1	0.033 (3)	0.044 (3)	0.034 (3)	0.002 (2)	0.021 (2)	−0.001 (2)
N2	0.045 (3)	0.039 (3)	0.043 (3)	−0.009 (2)	0.033 (3)	−0.001 (2)
N3	0.039 (3)	0.035 (3)	0.043 (3)	0.002 (2)	0.029 (2)	0.003 (2)
C1	0.053 (4)	0.059 (4)	0.040 (3)	−0.005 (3)	0.033 (3)	−0.002 (3)
C2	0.061 (5)	0.081 (6)	0.051 (4)	−0.013 (4)	0.037 (4)	−0.018 (4)
C3	0.051 (4)	0.089 (6)	0.046 (4)	−0.005 (4)	0.031 (4)	−0.023 (4)
C4	0.044 (4)	0.050 (4)	0.056 (4)	−0.007 (3)	0.031 (3)	−0.013 (3)
C5	0.030 (3)	0.045 (3)	0.040 (3)	0.005 (3)	0.020 (3)	−0.002 (3)
C6	0.033 (3)	0.039 (3)	0.039 (3)	0.002 (3)	0.021 (3)	−0.001 (3)
C7	0.040 (3)	0.046 (4)	0.054 (4)	−0.013 (3)	0.026 (3)	−0.010 (3)
C8	0.051 (4)	0.052 (4)	0.068 (5)	−0.011 (3)	0.040 (4)	0.002 (4)
C9	0.061 (4)	0.060 (4)	0.056 (4)	−0.010 (4)	0.045 (4)	0.005 (4)
C10	0.046 (4)	0.045 (3)	0.045 (3)	−0.003 (3)	0.035 (3)	0.004 (3)
C11	0.027 (3)	0.036 (3)	0.038 (3)	0.005 (2)	0.022 (3)	0.005 (3)
C12	0.057 (4)	0.055 (4)	0.043 (4)	−0.014 (4)	0.031 (4)	−0.003 (3)
C13	0.093 (6)	0.065 (5)	0.045 (4)	−0.033 (5)	0.041 (5)	−0.011 (4)
C14	0.121 (8)	0.071 (6)	0.065 (5)	−0.043 (6)	0.070 (6)	−0.036 (5)
C15	0.106 (7)	0.061 (5)	0.084 (6)	−0.014 (5)	0.082 (6)	−0.017 (5)
C16	0.072 (5)	0.042 (4)	0.068 (5)	−0.010 (3)	0.059 (4)	−0.007 (3)
C17	0.051 (4)	0.047 (4)	0.070 (4)	0.002 (3)	0.051 (4)	0.002 (3)
C18	0.081 (6)	0.045 (4)	0.108 (7)	0.003 (4)	0.081 (6)	−0.003 (5)
C19	0.057 (5)	0.058 (5)	0.113 (8)	0.023 (4)	0.060 (6)	0.024 (5)
C20	0.040 (4)	0.059 (5)	0.081 (6)	0.009 (4)	0.032 (4)	0.021 (4)
C21	0.040 (4)	0.050 (4)	0.065 (4)	−0.001 (3)	0.033 (4)	0.003 (4)
C22	0.040 (3)	0.037 (3)	0.062 (4)	0.000 (3)	0.040 (3)	0.005 (3)
C23	0.035 (3)	0.037 (3)	0.036 (3)	−0.007 (3)	0.024 (3)	−0.008 (3)
C24	0.047 (3)	0.032 (3)	0.041 (3)	−0.005 (3)	0.034 (3)	−0.003 (3)
C25	0.051 (4)	0.052 (4)	0.056 (4)	−0.007 (3)	0.037 (4)	−0.004 (3)
C26	0.090 (6)	0.051 (4)	0.091 (6)	−0.026 (5)	0.075 (6)	−0.017 (5)
C27	0.104 (7)	0.044 (4)	0.077 (5)	−0.013 (4)	0.073 (6)	−0.002 (4)
C28	0.087 (6)	0.057 (4)	0.057 (4)	0.007 (4)	0.053 (5)	0.013 (4)
C29	0.057 (4)	0.052 (4)	0.050 (4)	0.002 (3)	0.038 (4)	0.005 (3)
O3	0.036 (2)	0.031 (2)	0.092 (3)	0.0002 (17)	0.038 (2)	−0.017 (2)
C30	0.036 (2)	0.031 (2)	0.092 (3)	0.0002 (17)	0.038 (2)	−0.017 (2)
C30A	0.036 (2)	0.031 (2)	0.092 (3)	0.0002 (17)	0.038 (2)	−0.017 (2)

### Geometric parameters (Å, º)

**Table:**

Ir1—C22	1.991 (6)	C9—C10	1.367 (9)
Ir1—C11	2.016 (6)	C10—C11	1.397 (8)
Ir1—N1	2.035 (5)	C10—H10	0.9400
Ir1—N2	2.038 (5)	C12—C13	1.370 (10)
Ir1—N3	2.127 (5)	C12—H12	0.9400
Ir1—O2	2.207 (4)	C13—C14	1.383 (13)
F1—C7	1.371 (8)	C13—H13	0.9400
F2—C9	1.340 (15)	C14—C15	1.364 (13)
F2A—C9	1.33 (3)	C14—H14	0.9400
F3—C18	1.49 (4)	C15—C16	1.395 (10)
F3A—C18	1.21 (5)	C15—H15	0.9400
F4—C20	1.368 (9)	C16—C17	1.454 (10)
O1—C23	1.268 (7)	C17—C18	1.398 (10)
O2—H2A	0.854 (10)	C17—C22	1.429 (9)
O2—H2B	0.849 (10)	C18—C19	1.360 (12)
N1—C1	1.353 (8)	C19—C20	1.368 (12)
N1—C5	1.368 (8)	C19—H19	0.9400
N2—C12	1.353 (8)	C20—C21	1.374 (10)
N2—C16	1.353 (8)	C21—C22	1.383 (9)
N3—C23	1.309 (7)	C21—H21	0.9400
N3—H3	0.8700	C23—C24	1.504 (8)
C1—C2	1.382 (10)	C24—C29	1.379 (9)
C1—H1	0.9400	C24—C25	1.384 (9)
C2—C3	1.350 (12)	C25—C26	1.397 (10)
C2—H2	0.9400	C25—H25	0.9400
C3—C4	1.397 (10)	C26—C27	1.356 (12)
C3—H3A	0.9400	C26—H26	0.9400
C4—C5	1.380 (9)	C27—C28	1.382 (11)
C4—H4	0.9400	C27—H27	0.9400
C5—C6	1.470 (9)	C28—C29	1.388 (10)
C6—C7	1.392 (9)	C28—H28	0.9400
C6—C11	1.404 (8)	C29—H29	0.9400
C7—C8	1.379 (10)	O3—C30	1.387 (9)
C8—C9	1.374 (10)	O3—C30A	1.472 (9)
C8—H8	0.9400		
			
C22—Ir1—C11	92.5 (2)	C10—C11—Ir1	127.1 (5)
C22—Ir1—N1	97.2 (2)	C6—C11—Ir1	113.8 (4)
C11—Ir1—N1	80.3 (2)	N2—C12—C13	121.9 (8)
C22—Ir1—N2	80.8 (2)	N2—C12—H12	119.1
C11—Ir1—N2	95.9 (2)	C13—C12—H12	119.1
N1—Ir1—N2	175.72 (19)	C12—C13—C14	118.7 (8)
C22—Ir1—N3	89.3 (2)	C12—C13—H13	120.6
C11—Ir1—N3	175.3 (2)	C14—C13—H13	120.6
N1—Ir1—N3	95.12 (19)	C15—C14—C13	119.4 (7)
N2—Ir1—N3	88.66 (18)	C15—C14—H14	120.3
C22—Ir1—O2	174.2 (2)	C13—C14—H14	120.3
C11—Ir1—O2	90.00 (19)	C14—C15—C16	120.8 (8)
N1—Ir1—O2	88.38 (18)	C14—C15—H15	119.6
N2—Ir1—O2	93.72 (19)	C16—C15—H15	119.6
N3—Ir1—O2	88.65 (17)	N2—C16—C15	119.0 (7)
Ir1—O2—H2A	90 (5)	N2—C16—C17	113.2 (6)
Ir1—O2—H2B	92 (5)	C15—C16—C17	127.8 (7)
H2A—O2—H2B	95 (7)	C18—C17—C22	117.6 (7)
C1—N1—C5	118.7 (5)	C18—C17—C16	126.5 (7)
C1—N1—Ir1	124.4 (4)	C22—C17—C16	115.9 (6)
C5—N1—Ir1	116.8 (4)	F3A—C18—C19	112 (3)
C12—N2—C16	120.2 (6)	F3A—C18—C17	124 (3)
C12—N2—Ir1	123.3 (5)	C19—C18—C17	123.7 (8)
C16—N2—Ir1	116.5 (4)	F3A—C18—F3	11 (5)
C23—N3—Ir1	130.2 (4)	C19—C18—F3	121 (3)
C23—N3—H3	114.9	C17—C18—F3	115 (3)
Ir1—N3—H3	114.9	C18—C19—C20	116.3 (7)
N1—C1—C2	121.5 (7)	C18—C19—H19	121.9
N1—C1—H1	119.3	C20—C19—H19	121.9
C2—C1—H1	119.3	C19—C20—F4	117.6 (7)
C3—C2—C1	120.3 (7)	C19—C20—C21	124.5 (8)
C3—C2—H2	119.9	F4—C20—C21	117.9 (8)
C1—C2—H2	119.9	C20—C21—C22	118.8 (7)
C2—C3—C4	119.2 (7)	C20—C21—H21	120.6
C2—C3—H3A	120.4	C22—C21—H21	120.6
C4—C3—H3A	120.4	C21—C22—C17	119.1 (6)
C5—C4—C3	119.4 (7)	C21—C22—Ir1	127.5 (5)
C5—C4—H4	120.3	C17—C22—Ir1	113.4 (5)
C3—C4—H4	120.3	O1—C23—N3	122.5 (5)
N1—C5—C4	120.9 (6)	O1—C23—C24	117.1 (5)
N1—C5—C6	112.4 (5)	N3—C23—C24	120.4 (5)
C4—C5—C6	126.7 (6)	C29—C24—C25	118.9 (6)
C7—C6—C11	118.1 (6)	C29—C24—C23	122.1 (6)
C7—C6—C5	125.6 (6)	C25—C24—C23	119.0 (6)
C11—C6—C5	116.3 (5)	C24—C25—C26	120.5 (7)
F1—C7—C8	116.6 (6)	C24—C25—H25	119.8
F1—C7—C6	119.2 (6)	C26—C25—H25	119.8
C8—C7—C6	124.1 (6)	C27—C26—C25	119.8 (8)
C9—C8—C7	115.0 (6)	C27—C26—H26	120.1
C9—C8—H8	122.5	C25—C26—H26	120.1
C7—C8—H8	122.5	C26—C27—C28	120.6 (7)
F2A—C9—F2	6 (2)	C26—C27—H27	119.7
F2A—C9—C10	118.9 (16)	C28—C27—H27	119.7
F2—C9—C10	119.5 (8)	C27—C28—C29	119.7 (8)
F2A—C9—C8	116.3 (16)	C27—C28—H28	120.2
F2—C9—C8	115.9 (8)	C29—C28—H28	120.2
C10—C9—C8	124.6 (7)	C24—C29—C28	120.5 (7)
C9—C10—C11	119.1 (6)	C24—C29—H29	119.7
C9—C10—H10	120.4	C28—C29—H29	119.7
C11—C10—H10	120.4	C30—O3—C30A	44.6 (8)
C10—C11—C6	119.0 (5)	C30A^i^—C30A—O3	111 (2)
			
C22—Ir1—N1—C1	−86.8 (5)	O2—Ir1—N1—C1	91.6 (5)

Symmetry code: (i) −*x*+1, *y*, −*z*+1/2.

### Hydrogen-bond geometry (Å, º)

**Table:**

*D*—H···*A*	*D*—H	H···*A*	*D*···*A*	*D*—H···*A*
O2—H2*B*···N3	0.85 (1)	2.54 (6)	3.029 (7)	117 (6)
O2—H2*B*···O1	0.85 (1)	1.74 (3)	2.560 (6)	161 (7)
O2—H2*A*···N1	0.85 (1)	2.45 (6)	2.960 (6)	119 (6)
O2—H2*A*···O1^ii^	0.85 (1)	1.97 (4)	2.700 (7)	142 (6)

Symmetry code: (ii) −*x*+1/2, −*y*+3/2, −*z*.
